# Assessment of Mortality and Smoking Rates Before and After Reduction in Community-wide Prevention Programs in Rural Maine

**DOI:** 10.1001/jamanetworkopen.2019.5877

**Published:** 2019-06-14

**Authors:** Daniel K. Onion, Roderick E. Prior, N. Burgess Record, Sandra S. Record, Gerald R. Cayer, Christopher I. Amos, Thomas A. Pearson

**Affiliations:** 1 Maine-Dartmouth Family Medicine Residency, MaineGeneral Medical Center, Augusta; 2 Department of Community and Family Medicine, Geisel School of Medicine at Dartmouth, Hanover, New Hampshire; 3 Franklin Memorial Hospital, Farmington, Maine; 4 Lewis County General Hospital, Lowville, New York; 5 Department of Medicine, Baylor College of Medicine, Houston, Texas; 6 College of Medicine and Public Health and Health Professions, University of Florida, Gainesville

## Abstract

**Importance:**

It is unclear whether effective population-wide interventions that reduce risk factors and improve health result in sustained benefits to a community's health. If benefits do persist after a program is ended, interventions could be brief rather than maintained long term.

**Objective:**

To measure mortality and smoking rates in a rural community over decades before, during, and after prevention program reductions.

**Design, Setting, and Participants:**

This cross-sectional study compared smoking and mortality rates in a rural Maine county with other Maine counties over time by 5-year intervals. Multiple changes occurred between 2001 and 2015 in the physiological and behavioral risk factor reduction programs offered in the county. They included reductions in leadership, staff, institutional resources, data monitoring, and the programs themselves. Data were analyzed from May 2018 to March 2019.

**Intervention:**

Previous multifaceted interventions and outcome monitoring were withdrawn or diminished in the past decade.

**Main Outcomes and Measures:**

Smoking and age-adjusted mortality rates vs household income.

**Results:**

Reduced mortality rates in Franklin County in 1986 to 2005 reverted to those predicted by household incomes, relative to other Maine counties, by 2006 to 2015 (1986-1990 T score = −2.86 [*P* = .01] and 2001-2005 T score = −3.00 [*P* = .01] to 2006 to 2010 T score = −0.43 [*P* = .67] and 2011-2015 T score = −0.72 [*P* = .48]). Analysis of County Health Rankings data from 2010 to 2018 also showed that Franklin County’s outcomes have reverted to no better than predicted by socioeconomic status. The county’s T scores increased from −3.62 (*P* = .003) in 2010 to −0.41 (*P* = .69) in 2015 to 0.13 (*P* = .90) in 2018. Statewide association of income with mortality by analyses of variance showed that the *R*^2^ values have increased from the decades preceding 2000 (1976-1980, *R*^2^ = 0.21; *P* = .08; 1986-1990, *R*^2^ = 0.32; *P* = .02) to 2006 to 2010 (*R*^2^ = 0.73; *P* < .001) and 2011 to 2015 (*R*^2^ = 0.70; *P* < .001).

**Conclusions and Relevance:**

This study suggests that gains associated with population health interventions may be lost when the interventions are reduced. Adjusting outcome measures for socioeconomic status may allow quicker and more sensitive monitoring of intervention adequacy and success. The increasing trend of age-adjusted mortality in Maine and nationally to correlate inversely with incomes may warrant further community interventions, especially for poorer populations.

## Key Points

**Question:**

Do improved healthy behaviors and mortality in a rural county persist after community-wide interventions to mitigate behavioral and medical mortality risk factors have been ended or reduced?

**Findings:**

In this cross-sectional study of a rural Maine county, increasing mortality and smoking rates were observed within 5 to 10 years of program reductions. Also, mortality rates in Maine counties were more strongly associated with income in 2006 to 2015 than in previous decades.

**Meaning:**

This study suggests that there is a need for ongoing community population health assessments and prevention program adjustments to maintain reduced risk and positive outcomes.

## Teaser

This cross-sectional study estimates mortality and smoking rates in a rural county in Maine 5 to 10 years after reductions in a countywide set of interventions designed to address behavioral and medical risk factors.

## Introduction

Preventive interventions to reduce morbidity, mortality, and health care costs, especially from cardiovascular disease, have been tried in diverse communities.^1^ A few relatively brief, comprehensive, community-wide risk-reduction studies in various settings with nonintervention comparison populations have been reported.^2,3,4^ With the exception of the Finnish North Karelia program,^4^ those studies reported inconsistent results^5^ and often lacked sustained interventions or consistent engagement with local health care systems. Few studies have sustained interventions, documented preventive services, monitored changes in risk factors and behaviors, or measured any reductions in morbidity and mortality. And very few were in rural, socially disadvantaged communities,^6,7^ which typically lag behind metropolitan areas, especially in cardiovascular mortality improvements.^8,9^

Several previous reports^10,11,12,13^ described efforts to improve population health over 40 years in Franklin County, a low-income, rural, 97% white county in west central Maine. In the late 1960s, multiple local community groups identified improved health and health care as one of several remediable needs. A community action agency, a nonprofit medical group practice, and, later, the community hospital, initiated and coordinated their programs to improve health care quality and access and prevent disease. Our 2015 report^13^ described what this community collaboration accomplished over the decades during which intervention programs were established. That report tracked existing county vital statistics, rates of smoking cessation, and control of hypertension and high cholesterol up to 2010 and also extended prior mortality observations.^13^ The study also adjusted mortality and hospitalization rates by household income, a measure of socioeconomic status (SES). Those efforts were associated with coincident morbidity and mortality improvements when compared with all other Maine counties, especially when adjusted for income.

Since that 2015 report, many of the interventions to improve health and reduce risk factors described there have been reduced or ended. How long improvements might last after such programs’ reductions or withdrawals is unknown. Whether such population-wide interventions can be diminished after a time without detriment to a community's health is unclear. If they can, program interventions could be temporary, reduced, or intermittent. This article describes the reduction in interventions in Franklin County over a recent 10-year period and associated increases in mortality and smoking rates. Continuing and increased associations of household income and mortality in all Maine counties in recent decades were also documented.

## Methods

### Study Design

This was a serial cross-sectional study of a rural county population over time. It measured countywide mortality and smoking rates compared with the 15 other Maine counties by 5-year intervals and examined those outcomes’ association with countywide household income before, during, and after community intervention reductions. This study also obtained data from the County Health Rankings project^14^ as an additional measure of the effect of SES on health outcomes and the status of Franklin County as an outlier to that association. This analysis followed methods used in a study by the Institute for Healthcare Improvement of that association.^15^ The University of Southern Maine institutional review board determined that the study participant population did not fall under the definition of human subjects and, thus, was exempt from further review. This study followed the Strengthening the Reporting of Observational Studies in Epidemiology (STROBE) reporting guideline.

### Data and Sources

Age-adjusted mortality per 100 000 population and income data on Franklin County and all 15 other Maine counties’ residents for 5-year periods as far back as 1971, as well as smoking rates between 1996 and 2015 (the period for which such data were available), were collected and reanalyzed with new 2011 to 2015 data from Centers for Disease Control and Prevention Compressed Mortality Files,^16^ US Census Bureau American Community Survey,^17^ and the Centers for Disease Control and Prevention Behavioral Risk Factor Surveillance System.^18^ Maine county health outcomes and health factors data were downloaded from County Health Rankings (CHR).^14^

### Statistical Analysis

To adjust for an increasing association between income and mortality as well as smoking, linear regression and analysis of variance were performed on age-adjusted mortality vs median household income, smoking vs income, and CHR health outcomes vs SES scores. The CHR^14^ data were analyzed for each available year, 2011 to 2018, following the methods of Whittington et al.^15^

Standardized observed-minus-expected values (T scores) were calculated for smoking rates, mortality rates, or CHR outcomes compared with those predicted by all Maine counties’ incomes or CHR SES, respectively. Scatter diagrams were plotted with calculated *R*^2^ values (the square of Pearson correlation coefficients) showing the strength of the associations. Statistical *P* values and confidence intervals were calculated at a 5%, 2-tailed level and 14 *df*. All analyses of old and new data were done from May 2018 to March 2019.

### Additional Data and Evaluations

A conference was held in September 2017 to present, discuss, and assess these new data. Participants included the local authors of our previous *JAMA* article,^13^ local stakeholders (medical, community health, referral hospital, public health nursing, and community action agency), along with several Maine population health experts, funders, and a former state economist.

## Results

### Demographic Characteristics

The population of Franklin County grew by 30% between 1970 and 1990, but little since, unlike the state as a whole, where estimated growth has only slowed since 2010 (Table 1). Maine has consistently ranked as one of the oldest US states by median and/or mean age over recent decades. Franklin County has become older and poorer than the average Maine county over that same period. Both Franklin County and Maine populations have declined slightly from 99% white in 1970 to approximately 95% most recently. Population to primary care physician ratios halved between 1970 and 2015, as ratios for Franklin County and all of Maine remained comparable.

**Table 1.** Franklin County and Maine Demographic Characteristics, 1970 to 2015^a^

Characteristic	1970		1980		1990		2000		2010		2015	
	FC	ME	FC	ME	FC	ME	FC	ME	FC	ME	FC	ME
Population, No.	22 387	994 538	27 101	1 125 413	28 997	1 227 806	29 467	1 274 923	30 768	1 328 361	30 039	1 329 453
Population per sq mile, No.	13	32	16	37	17	40	17	42	18	43	18	43
White race/ethnicity, %	99.9	99.3	99.5	99.0	99.2	98.4	97.6	96.5	97.7	95.6	97.0	95.0
Age												
Median, y	26.8	29.1	29.7	30.5	32.9	33.9	38.2	38.6	43.5	42.8	45.8	44.5
Population <18 y, %	35.3	34.9	34.0	32.3	26.2	25.2	23.5	23.6	19.6	20.6	18.3	19.3
Population >65 y, %	10.8	11.6	12.0	12.6	12.3	13.3	14.2	14.4	17.2	16.0	19.9	18.8
Adults aged >25 y without a high school diploma, %	42.8	45.3.7	29.7	31.3	20.3	21.2	14.8	14.6	12.3	10.2	7.8	8.4
Persons in poverty, %	11.6	13.6	12.8	13.0	12.5	10.8	14.6	10.9	16.7	12.6	14.8	13.9
Ratio of population to primary care physicians	1870	1900^b^	1500^b^	1550^b^	1250	1250	951	1035	853	952	901	935

Abbreviations: FC, Franklin County; ME, Maine.

^a^ Total population figures are from the Centers for Disease Control and Prevention Compressed Mortality File.^16^ All age, race, education, and poverty levels were obtained from the decennial US Census for years 1970, 1980, 1990, 2000, and 2010 in January to March 2013, supplemented with 2000 to 2015 data from the US Census Bureau’s American Factfinder.

^b^ Values are approximate.

### Community Interventions

Multiple changes occurred between 2001 and 2015 in the physiological and behavioral risk factor reduction programs run by the Franklin County Healthy Communities Coalition and the Franklin Cardiovascular Health Program. They included reductions in leadership, staff, institutional resources, data monitoring, and the programs themselves (eTable 1 in the Supplement).

### Mortality Rates Increased

**Figure 1.**
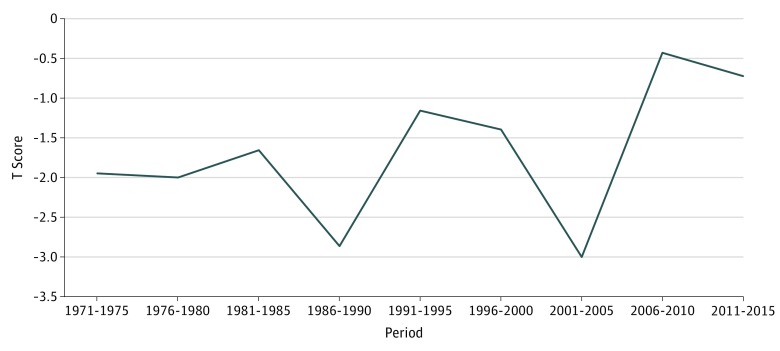
T Scores for Mortality vs Income, Franklin County, Maine, 1971 to 2015 Standardized observed Franklin County mortality minus expected mortality rate predicted by linear regression for 5-year periods from 1971 to 2015. T scores are the number of standard deviations of the Franklin County observed mortality rate minus the income-predicted score. Negative T scores represent better mortality outcomes in Franklin County than predicted by income. The previous 1990 to 1995 increases toward the income-predicted baseline were also associated with community prevention programmatic cutbacks,^12^ as is the more recent and dramatic relative mortality increase reported between 2001 and 2015 here.

**Figure 2.**
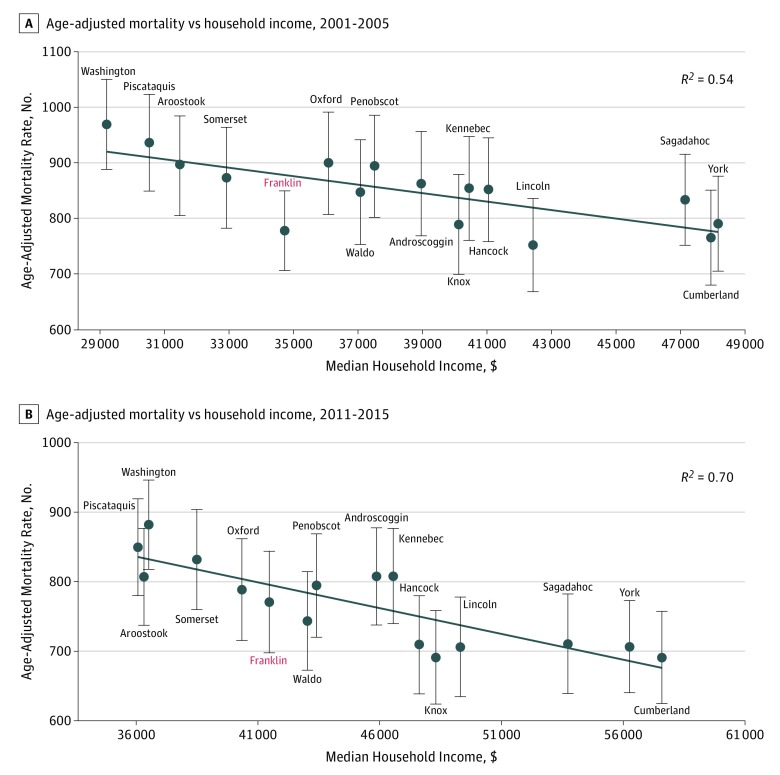
Maine County Age-Adjusted Mortality vs Household Income Maine county age-adjusted mortality vs median household income with a simple linear regression line shown. Error bars represent 95% confidence intervals for each county. Confidence intervals that cross the regression line are not statistically significant. The included analysis of variance *R*^2^ indicates the proportion of the variance among the data points associated with income. These 2 panels show Franklin County’s reversion by 2010 to 2015 from a favorable outlier relative to other Maine counties in 2001 to 2005, as is reflected in Figure 1.

Since 1970, Franklin County age-adjusted mortality T scores have been consistently lower than predicted by household income when compared with the other Maine counties (Table 2, Figure 1, and Figure 2). Franklin County was a significant outlier in 1986 to 1990 (T score = −2.86; *P* = .01) and 2001 to 2005 (T score = −3.00; *P* = .01). The previous 1990 to 1995 increases toward the income-predicted baseline were also associated with community prevention programmatic cutbacks,^12^ as is the more recent and dramatic relative mortality increase reported between 2001 and 2015. However, during the most recent 5-year intervals, 2006 to 2010 and 2011 to 2015, the county’s T scores have increased toward the regression line as mortality rates approached those predicted by Maine average county household income more closely than at any time in the past 45 years (2006-2010 T score = −0.43; *P* = .67 and 2011-2015 T score = 0.72; *P* = .48).

**Table 2.** Franklin County and All Other Maine County Mortality and Income Data, in 5-Year Periods, 1971 to 2015

Period	Franklin County				All Maine Counties	
	Age-Adjusted Mortality Rate, No./100 000 Population	Median Household Income, $	Mortality vs Income T Score	*P* Value for T Score	*R*^2^	*P* Value for *R*^2^
1971-1975	1105.8	8693	−1.95	.07	.24	.05
1976-1980	914.8	13 500	−2.00	.07	.21	.08
1981-1985	925.8	17 873	−1.66	.12	.06	.37
1986-1990	893.2	23 339	−2.86	.01	.32	.02
1991-1995	880.3	27 243	−1.16	.27	.40	.008
1996-2000	856.6	30 756	−1.40	.18	.57	.001
2001-2005	778.1	34 727	−3.00	.01	.54	.001
2006-2010	793.3	38 811	−0.43	.67	.73	<.001
2011-2015	770.8	41 538	−0.72	.48	.70	<.001

Comparable data from CHR and their analysis of CHR outcomes vs SES factors support these results, showing a progressive worsening in Franklin County outcomes, adjusted for SES, compared with all other Maine counties from 2010 (T score = −3.62; *P* = .003) to 2015 (T score = −0.41; *P* = .69) to 2018 (T score = 0.13; *P* = .90) (eFigure 1 in the Supplement).

### Smoking Rates Increased

In 1996 to 2000, Franklin County income-adjusted smoking rates made the county an outlier (T score= −3.31, *P* = .005). Thereafter, they gradually increased, and by 2011 to 2015, were similar to rates predicted by Maine county household incomes (T score = −0.33; *P* = .75) (eFigure 2 in the Supplement).

### Association Between Maine County Income and Mortality Increase Statewide

**Figure 3.**
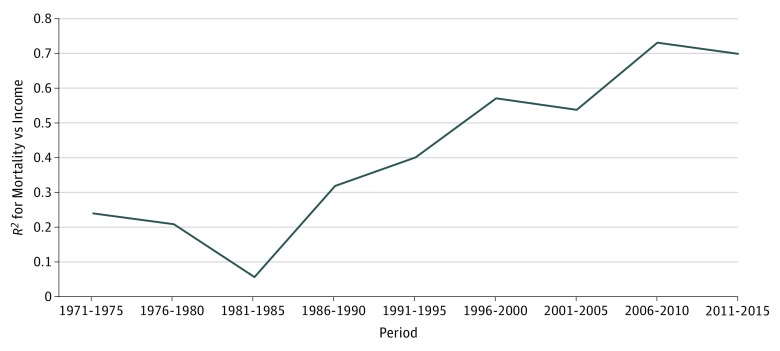
*R*^2^ Value of Maine County Age-Adjusted Mortality vs Median Household Income for 5-Year Periods, 1976 to 2015 Statewide *R*^2^ associations of income with mortality show an increasing inverse association of mortality with income over time, consistent with similar national observations, especially since 2000. *R*^2^ is obtained by analysis of variance and varies between 0 and 1 or 100%. It represents the proportion of the variance among Maine counties of mortality rates associated with county household income.

Statewide association of county income with county mortality by analysis of variance *R*^2^ has increased. Values have increased over the last 4 decades (1976-1980: *R*^2^ = 0.21 [*P* = .08]; 1986-1990: *R*^2^ = 0.32 [*P* = .02]; 2006-2010: *R*^2^ = 0.73 [*P* < .001]; and 2011-2015: *R*^2^ = 0.70 [*P* < .001]) (Figure 3), as did smoking rates, to a lesser degree (eFigure 3 in the Supplement).

### Stakeholders’ Conference

The local participants found Franklin County's relatively higher mortality and smoking rates to be consistent with their experience (eTable 2 in the Supplement). They cited the most likely causes to be loss of individual and institutional leadership in the community (medical, hospital, social, and business and industry), cessation of many component intervention programs, and a discontinuation of individual and population risk factor monitoring with likely consequent program effectiveness decline.

## Discussion

In this serial cross-sectional study of all residents in a rural Maine county, access to health care and risk factor improvement programs were associated with better outcomes for 40 years,^13^ but those appear to have had recent declines relative to other Maine counties, as demonstrated by the worsening mortality rates reported in this article. Stakeholders’ conference minutes cited most likely causes to be loss of individual and institutional leadership in the community (medical, hospital, social, and business and industry), cessation of many intervention programs’ components, and a discontinuation of individual and population risk factor monitoring with likely consequent program effectiveness decline. Perhaps the most critical loss was the cessation of computer entry systems, reversion to paper data monitoring, and associated inability to reliably measure individual and population outcomes against past results and future targets.

That program cutbacks may have contributed to deteriorating outcomes over recent decades is concerning but not surprising. Ongoing iterative population-based data gathering about process intermediate outcomes (like smoking), and ultimate outcomes (like mortality) is likely important for determining program adjustments, as it is in a wide range of medical interventions. Such population monitoring had been largely abandoned in Franklin County by 2015.

A recent study^19^ reported associations between increased mortality and higher population to primary care physician ratios. However, such explanations of our mortality findings seem unlikely because Franklin County’s ratios have been close to state averages over the decades and improved somewhat in 2015.

Most importantly, these observations suggest that health benefits persist for 10 years or less after effective community-wide health interventions have stopped or diminished. In fact, earlier studies in Franklin County found 2 similar, although less drastic, increases in overall mortality with previous community intervention reductions, and a lag of only ±2 years, when monitored yearly.^12^ Evidence-based community interventions in a low-income rural county can be associated with outcomes as good as or better than those in higher-income counties in Maine, but now we would add the caveat that ongoing maintenance of such programs is likely crucial for continuing benefits.

Similar increases in smoking rates, relative to other Maine counties, suggest a partial mechanism for the increased mortality. The Franklin County Healthy Communities Coalition has recently increased staff efforts, offering hope that they may once again succeed in rejuvenating their programs’ effectiveness, as has been done before.

Maine county mortality rates have been increasingly associated with lower county incomes over the past decades. A similar trend, to a lesser degree, is also apparent for smoking rates vs income. These findings are consistent with the worsening of health and mortality in US rural white populations, like that of Franklin County, detected nationally beginning in 2000.^20,21,22^ These populations have stagnant overall mortality rates, which are due in part to their cardiovascular mortality rates not continuing to improve, unlike other segments of the US population.^23^

Finally, adjusting outcomes like mortality against income seems to provide a more sensitive way to identify and monitor vulnerable rural populations than simple county-to-state average comparisons, as we have previously shown.^13^ Effective community medicine interventions may thus be analogous to clinical care management strategies of intensive supervision and support for high-risk patients with chronic disease in the United States.^24^

### Strengths

The analysis of the CHR data adds further support to the findings, as do recent Maine press reports^25^ of the 2019 CHR Franklin County data; those suggest further deterioration in health outcomes as well. This decades-long, geographically defined population study of a small, rural, predominantly white US county is unique, to our knowledge, and may shed light on and offer hope for addressing the recent reported worsening health outcomes in that segment of the US population.

### Limitations

This study has limitations. Counties, like Franklin, that support and adopt new public health interventions early on may appear to have worsened in comparison with their national and state peers when, in fact, other counties simply caught up. Franklin County did have absolute worsening mortality rates in 1990 to 1995 and 2010 to 2015, while the statewide mortality continued to decrease during both periods. Thus, catch-up by other Maine counties, as well as absolute decrements in Franklin County mortality rates, both may have played a role in Franklin falling behind others.

Our findings are limited by this study being a serial cross-sectional study of outcomes over time, not a randomized trial; associations do not necessarily mean causation. However, the consistency of the evidence suggests possible causal relationships. Favorable outcomes associated with active programmatic interventions lasted several decades. Their reversions to nearly statewide predicted rates, as those intervention programs regressed over 5 to 10 years, contributes further to a chain of evidence.

Methodologic changes in the national Behavioral Risk Factor Surveillance System telephone data gathering methods were made in the 2011 to 2015 period,^26^ when mobile phones began to be surveyed in addition to landlines. Absolute increases in smoking rates were reported in all Maine counties after that, suggesting that individuals who predominantly use mobile phones were more likely to smoke. Such observations confirm both the Behavioral Risk Factor Surveillance System’s and CHR’s reservations about using these data for sequential time comparisons because of those and other changing survey methods. However, T scores (standardized mortality rates), as supplied here, still allow relative county comparisons.

## Conclusions

Decades of relative gains in this rural white population's health were lost within 5 to 10 years of cutbacks of leadership and other programmatic support. Outcome measures, adjusted for SES, may allow quicker and more sensitive monitoring of intervention adequacy and success. The increasing trend of age-adjusted mortality in Maine and nationally to correlate inversely with incomes has been and could be addressed further with community interventions directed especially at poorer populations.

## Notes

Supplement. **eTable 1.** Changes in Franklin County Community Health Programs, 2000-2015 **eTable 2.** Minutes of September 6, 2017, Local Conference, Franklin Memorial Hospital, Farmington, ME **eFigure 1.** Maine Data From the County Health Rankings Annual Reports, 2010-2018 **eFigure 2.** Standardized Actual Versus Income—Adjusted Expected Smoking Rates (T-Scores) in Franklin County vs. Rest of Maine Counties, 1996-2015 **eFigure 3.** Maine County Smoking Rates vs Income; Association *R*^2^ Value
