# Error in Funding/Support

**DOI:** 10.1001/jamanetworkopen.2019.9340

**Published:** 2019-07-24

In the Original Investigation titled “Assessment of Mortality and Smoking Rates Before and After Reduction in Community-wide Prevention Programs in Rural Maine,”^1^ published June 14, 2019, there was an error in the Funding/Support section in Article Information. A grant from the Cancer Prevention Research Institute of Texas (RR170048) to Dr Amos was omitted. This article has been corrected.^1^
