# Supplementary material for: Assessment of Mortality and Smoking Rates Before and After Reduction in Community-wide Prevention Programs in Rural Maine
Source: JAMA Netw Open. 2019 Jun 14;2(6):e195877. doi: 10.1001/jamanetworkopen.2019.5877 (PMC6575143; doi:10.1001/jamanetworkopen.2019.5877)
Supplement: Supplement. — eTable 1. Changes in Franklin County Community Health Programs, 2000-2015 eTable 2. Minutes of September 6, 2017, Local Conference, Franklin Memorial Hospital, Farmington, ME eFigure 1. Maine Data From the County Health Rankings Annual Reports, 2010-2018 eFigure 2. Standardized Actual Versus Income—Adjusted Expected Smoking Rates (T-Scores) in Franklin County vs. Rest of Maine Counties, 1996-2015 eFigure 3. Maine County Smoking Rates vs Income; Association R2 Value [file jamanetwopen-2-e195877-s001.pdf]

Supplementary Online Content

Onion DK, Prior RE, Record NB, et al. Assessment of mortality and smoking rates before and after reduction in community-wide prevention programs in rural Maine. *JAMA Netw Open*. 2019;2(6):e195877. doi:10.1001/jamanetworkopen.2019.5877

eTable 1. Changes in Franklin County Community Health Programs, 2000-2015
eTable 2. Minutes of September 6, 2017, Local Conference, Franklin Memorial Hospital, Farmington, ME
eFigure 1. Maine Data From the County Health Rankings Annual Reports, 2010-2018
eFigure 2. Standardized Actual Versus Income—Adjusted Expected Smoking Rates (T-Scores) in Franklin County vs. Rest of Maine Counties, 1996-2015
eFigure 3. Maine County Smoking Rates vs Income; Association R^2 Value

This supplementary material has been provided by the authors to give readers additional information about their work.

© 2019 Onion DK et al. *JAMA Network Open.*

eTable 1. CHANGES IN FRANKLIN COUNTY COMMUNITY HEALTH PROGRAMS, 2000-2015

Years	Leadership	Staff	Institutional Resources	Data monitoring	Programs
2000-<br><br>2004	2000-2006: Continuing community health needs assessments and improvements with community "Visioning sessions".<br><br>2000+: Increasing difficulty recruiting leaders, especially physicians interested in community engagement approaches.<br><br>2004: Both FCHP medical and nursing directors redeployed to other hospital departments reducing commitment of time available for community engagement	2000: Franklin Cardiovascular Health Program (FCHP) peak staffing<br><br>2002: FCHP staff reduced x50%<br><br>2004: Loss of more FCHP staff to FCHN medical practices	2000: Peak FCHP grant funding.<br>New mobile screening van<br>State-wide dissemination of Franklin Co methods with tobacco settlement monies by Maine Governor King<br><br>2000-2006: hospital finances tighter; net collections down from 83% to 47%.<br><br>2003: FCHN re-established in-house mental and contracted dental health services with negotiated access for low-income patients (a "visioning" outcome)<br><br>2004: FCHP grant funding diminishes from private, state, and federal sources	2000: Effective computerized client tracking and data monitoring systems (CDMS) in place and working at FCHP and Healthy Community Coalition (HCC)	2000-2004: Extensive worksite, school, university, hospital BP/cholesterol screening/ coaching/referral/education systems; 2-3000 individual contacts/yr<br>•Hospital primary care nursing support of chronic disease (HT, cholesterol, diabetes) management
2005-<br><br>2009	2005: New hospital COO/EVP with MPH and strong community medicine interests arrives (leaves in 2017)<br><br>2008: Hospital CEO, long-time community medicine advocate, retires after 13 years<br><br>2009: New CEO from south Texas employed by hospital board with major charge to improve hospital finances; expressed and manifested little support for the community health-based programs' approach	2007: Key FCHP/HCC grant writer and staff member left for jobs elsewhere	2005+: Initial state-wide community health funding from tobacco settlement, subsequently eroded by lack of effective monitoring and diversion of monies elsewhere in state government<br><br>2008-New Medical Arts building completed (significant organizational overhead incurred)	2006+: CDMS maintenance and development moved out of FCHN building<br><br>2009: EMR installed in PCP offices; prior links to CDMS ended; limited utilization of patient-specific reports scanned into EMR	2005-2009: HCC continued some screening of risk factors but paper records only, with no follow-up, tracking or comprehensive overview.

© 2019 Onion DK et al. *JAMA Network Open.*

	2009+: Further difficulty recruiting physicians. Physician community medicine leadership diminished and less community engagement.<br><br>2009: Community health needs assessment conferences truncated and more institutionally- than community-driven.				
2010-<br><br>2015	2010+: Both FCHP medical and nursing directors retired<br><br>2010+: Diminished focus on FCHN senior staff maintaining active engagement with community organizations<br><br>2011-Hospital medical director, long-time advocate of community medicine, retired<br><br>2015: New CEO left; hospital integrated into and administered by a tertiary care system based 75 miles away.		2014: All HCC staff moved out of central hospital to peripheral space<br><br>2014: Hospital medical library, used by both clinicians and community, closed. Community use of classrooms restricted<br><br>2014+: UMF community health program loses departmental status; reduced number of administrative internships at hospital<br><br>2015: Hospital large conference room use by outside groups curtailed as a cost savings and liability risk	2010: CDMS product development, enhancements, and support reduced<br><br>2012+: HCC ended data entry into CDMS; reversion to paper records.	2011-HCC reduced van visit frequency and reduced feedback to PCPs, resulting in incomplete data of lesser value<br><br>2014: Hospital stopped accepting "contributed-labor" payment of medical bills<br><br>2015: Many programs for low income patients reduced or stopped, but continued help with free drugs applications<br><br>2015: Smoking reduction programs markedly reduced from peak activity just after 2000

Table 1. Abbreviations:
CDMS (Computerized Data Management Systems,
dedicated to community health preventive programs)
CEO (Chief Executive Officer)
COO (Chief Operating Officer)
EMR (Electronic Medical Record)
EVP (Executive Vice President)
FCHN (Franklin Community Health Network)
FCHP (Franklin Cardiovascular Health Program)
HCC (Healthy Communities Coalition)
RVU (Relative Value Units)
UMF (University of Maine Farmington

© 2019 Onion DK et al. *JAMA Network Open.*

eTable 2: Minutes of September 6, 2017 Local Conference, Franklin Memorial Hospital, Farmington, ME

FRANKLIN CO THEN AND NOW: Health Outcomes vs Socioeconomic Factors

Agenda Item	Discussion and Conclusions
Introductions	Dr. Dan Onion welcomed everyone, and introduced himself and each of the 24 participants
Presentations	*Dan Onion: Health Intermediate and Ultimate Outcomes Over Time in Franklin County, 1960-2015*<br>*Rod Prior: Variations in Health in the US, Outlier U.S. Counties Across U.S. Geographies and Across Time,*
Group Discussions: **"Is there a significant change in Franklin Co. and if so why?"**	The participants were split into two groups<br><br>Group A decided that there was a significant change. Possible reasons why include:<br>• decrease in social capital<br>• diminishing community infrastructure<br>• lack in leadership support<br>• RHA is no longer present<br>• Funding cuts to HCC<br>• Hospital CEO left 2010<br>• Grant writer MPH and leader left 2009<br>• FCHP med director leaves 2013-<br>• Score Health CDMS<br>• RVU productivity measurements push hurts community med work<br>• ACO may distort broader population health commitments<br>• UMF community health program diminished<br>• Corporatization of big local employers: the hospital, banks, local ski mountain, UMF as occurred. Thus leaderships are no longer here or willing/able to serve on health boards of directors.<br>• Rightward political shift hits on property tax, elderly, schools, social supports<br>• Deindustrialization of rim counties in Maine is a huge problem<br>• With fewer childbearing females/couples and other working age adults the middle years have been taken out of age distribution, possibly distorting the data.<br>• Franklin County income and education may be influenced by census counting of out-of-staters<br>• Is Franklin County "better" because of the ski and university systems as other industries decrease?<br>• An aid to these questions might be to quantitate jobs by employer type?<br><br>Group B decided that health behaviors have definitely gotten worse in Franklin Co. Local data on obesity, for example, but not sure if they have gotten worse at a higher rate than the rest of the state.

© 2019 Onion DK et al. *JAMA Network Open.*

	Possible reasons why include:<br>• Impact of great recession? (2008-2016?)<br>• Medicaid squeeze<br>• Lack of funding (local, regional, state, national)<br>• Maybe not much impact because Franklin Co. is so poor, but worksites could no longer afford FCHP programs.<br>• Franklin Co. interventions started declining prior to 2008 and continued declining over time.<br>• Big business/mills closed, difficult to find large groups of people<br>• Commitment of medical community to public/community health has declined with changes in medical practice.<br>• Quality of care in primary care visit has changed, PCPs tend to be more transient (do "their time" and move away)<br>• Institutional leadership is important<br>The two groups came together to share what each had discussed. In addition to the thoughts above, the following observations were mentioned:<br>• There was a huge focus on CVD prevention, everyone focused on it in Franklin Co.; CVD efforts are more diffuse now.<br>• Maine moved from the state with the 6th lowest (1990) uninsured rate to 24th (2016)<br>• Community Health Workers (CHWs) are a really important missing piece now<br>• Social assistance/community involvement is lacking<br>• We must be more politically outgoing and loud, advocating for/demanding public health and social supports
**Agenda Item**	**Discussion and Conclusions**
Panel Discussion: **"Speculation and discussion of possible renewed and/or new prioritized interventions potentially helpful for future Franklin Co health outcomes"**,	A Panel discussion with audience participation discussed next steps. The panelists were a tertiary care hospital community medicine leader, a Maine community medicine foundation project officer, and a University of Southern Maine health care research scientist with conference audience participation. The following ideas were expressed:<br><br>• -Move/do something, even if we do not have all the data.<br>• -Collaborate with adjacent Oxford Co.<br>• -PCPs should be all involved in population health<br>• -CHW and community involvement is key<br>• -Going to where the people are, not waiting for them to show up at the hospital<br>• -ACOs are where the work can continue<br>• -Get political<br>• -Share the Franklin Co. success story with the nation – can it be replicated?<br>• -Keep in mind rural vs. urban disparities<br>• -"Health care" is a toxic word these days<br>• -Take the focus away from "health care", and move toward addressing social determinants of health (supporting work of CAP agencies)

© 2019 Onion DK et al. *JAMA Network Open.*

	• -PCPs are already limited in their time with patients. Maybe we should move toward a social model (public, private, volunteer, local decision-making) and away from a medical model<br>• --Where does the money come from to pay for this? Possibly from revenue funding, adjustment to school funding formula, sales tax, income tax?<br>• --Attract immigrant workers<br>• --There are billing models (PCP and behavioral health); why not PCP and social services?<br>• -WMCA has a two-generation approach to poverty, in which they work with the family as a unit (ex. Help with childcare, and college expenses for mother). Maine Sen. Susan Collins is supportive of this approach-maybe reach out to her? HCC began out of the recognition of the two generation need.<br>• -Maybe set a goal to "reduce poverty by half in ten years", for example, and begin a study<br>- Example of effort underway – United Way of Greater Portland has an initiative (Thrive 2027) that addresses social determinants of health, decreasing years of life lost in Cumberland Co. with strategies to decrease suicides and decrease opiate use.<br>• -Is there a community health nexus in Franklin Co. already, or does one need to be re-created?<br>• -Yes, HCC is present, and it could be improved with more community input and involvement<br>-We must be able to track data, show outcomes.
**Agenda Item**	**Discussion and Conclusions**
Final Thoughts	Final Question and Thoughts:<br>• Should we (a) replicate what has already been done, or (b) focus on social determinants of health/comprehensive cross sector look? Go with option (b), and switch to singular focus = Poverty.<br>• Convene community groups<br>• Share this information, get more people involved, go to where the people are<br>• Rekindle past work, but change focus to poverty utilizing navigators<br>• Work in conjunction with what came out of *Donna Beegle's Poverty Training and Workshop?*

eTable 2. Participant Titles:
CEO, Lewis County General Hospital and former EVP/COO, FMH
Research Professor and Director, Maine Rural Health Research Center, Muskie School, Univ. Southern Maine
Director, Data Reporting and Evaluation, Center for Health Improvement, MaineHealth
Senior Vice President, Community Health, MaineHealth
Retired General Surgeon (50 yr in Franklin Co) and founder of RHA; former hospital VP for Medical Affairs
Former Director, WMCA
Senior Program Director HCC
Public Health Consultant
Executive Director, WMCA
Senior Program Officer, Maine Health Access Foundation (MeHAF)
Former UMF Professor of Economics, living; recent State Economist, now consulting

© 2019 Onion DK et al. *JAMA Network Open.*

COO, Franklin Memorial Hospital
Pediatrician/ Community Medicine, Franklin Memorial Hospital
President and CEO, MeHAF
Executive Director, Healthy Community Coalition
Senior Program Officer, The Bingham Program
VP for Clinical Affairs; Director, Center for Health Innovation and Interim
VP for Research, Univ. New England
Staff Nurse, Healthy Communities Coalition
VP Medical Affairs, FMH
Family Medicine Residency Director-emeritus; Prof Community + Family
Medicine, Geisel School of Medicine at Dartmouth l, Former Franklin Area Health Plan Medical Director
Franklin Co. internist, former hospitalist, occupational health physician, medical director, FMH CIO
Founding and long-time Medical director of FCHP, and practicing internist
Former nurse manager of the FHCP
Trustee, The Betterment Fund

eTable 2. Abbreviations:
ACO (Accountable Care Organization)
CDMS (Computerized Data Management Systems)
CEO (Chief Executive Officer)
CHW (Community Health Worker)
CIO (Chief Information Officer)
CVD (Cardiovascular Disease)
FCHP (Franklin Cardiovascular Health Program)
FMH (Franklin Memorial Hospital)
HCC (Healthy Community Coalition)
MeHAF (Maine Health Access Foundation)
PCP (Primary Care Physician/Clinician)
RHA (Rural Health Associates)
RVU (Relative Value Unit)
UMF (University of Maine Farmington)
VP (Vice President)
WMCA (Western Maine Community Agency

© 2019 Onion DK et al. *JAMA Network Open.*

eFigure 1. Maine Data From the County Health Rankings Annual Reports, 2010-2018

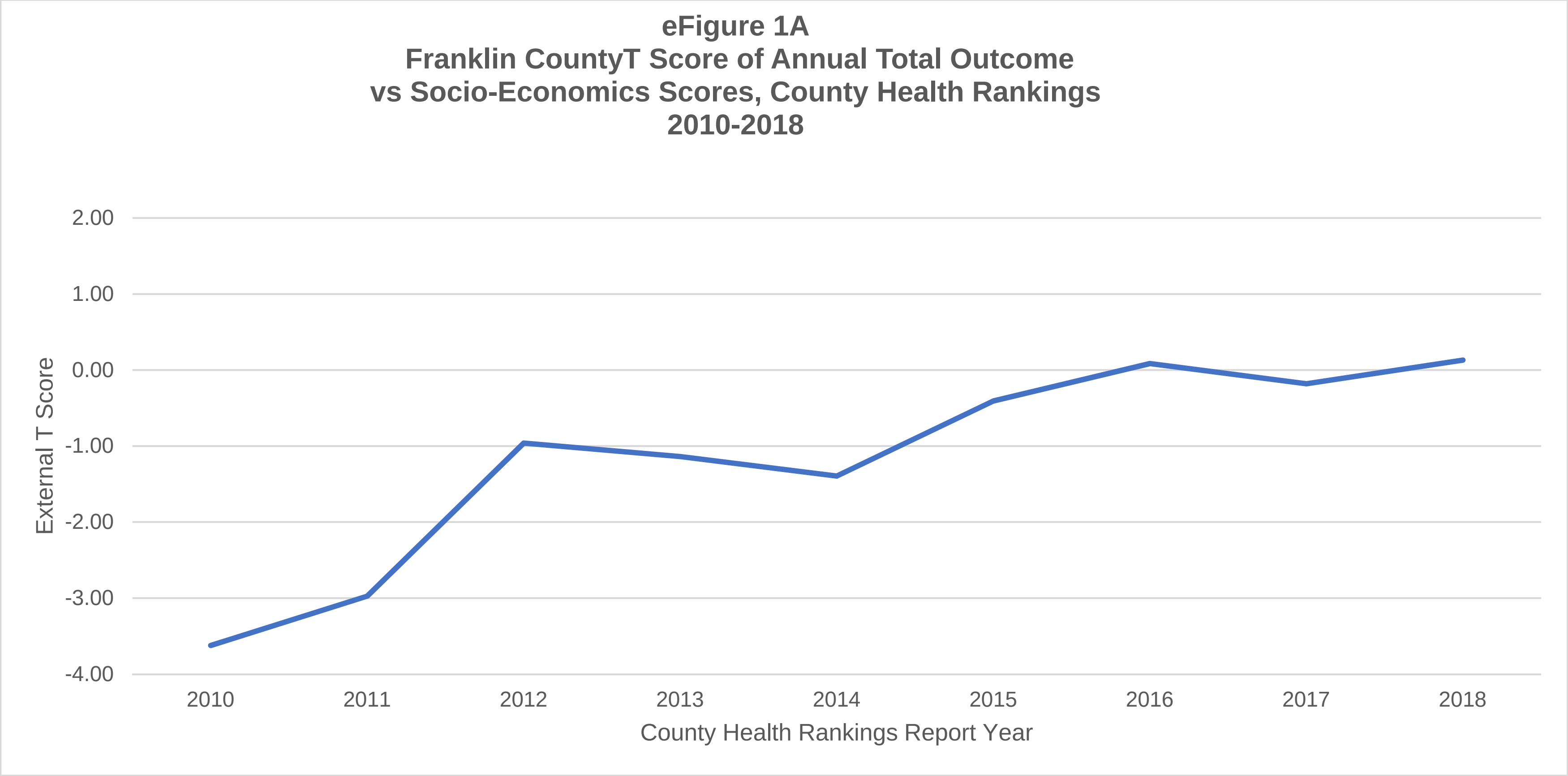

Legend for eFigure 1A: Maine Data from the County Health Rankings annual reports, 2010-2018. CHR data reflects various county health outcomes and factors known to be associated with health. Of all CHR Health Factor Scores, the Total Socioeconomic Score has the strongest correlation with Total Outcomes.

© 2019 Onion DK et al. *JAMA Network Open.*

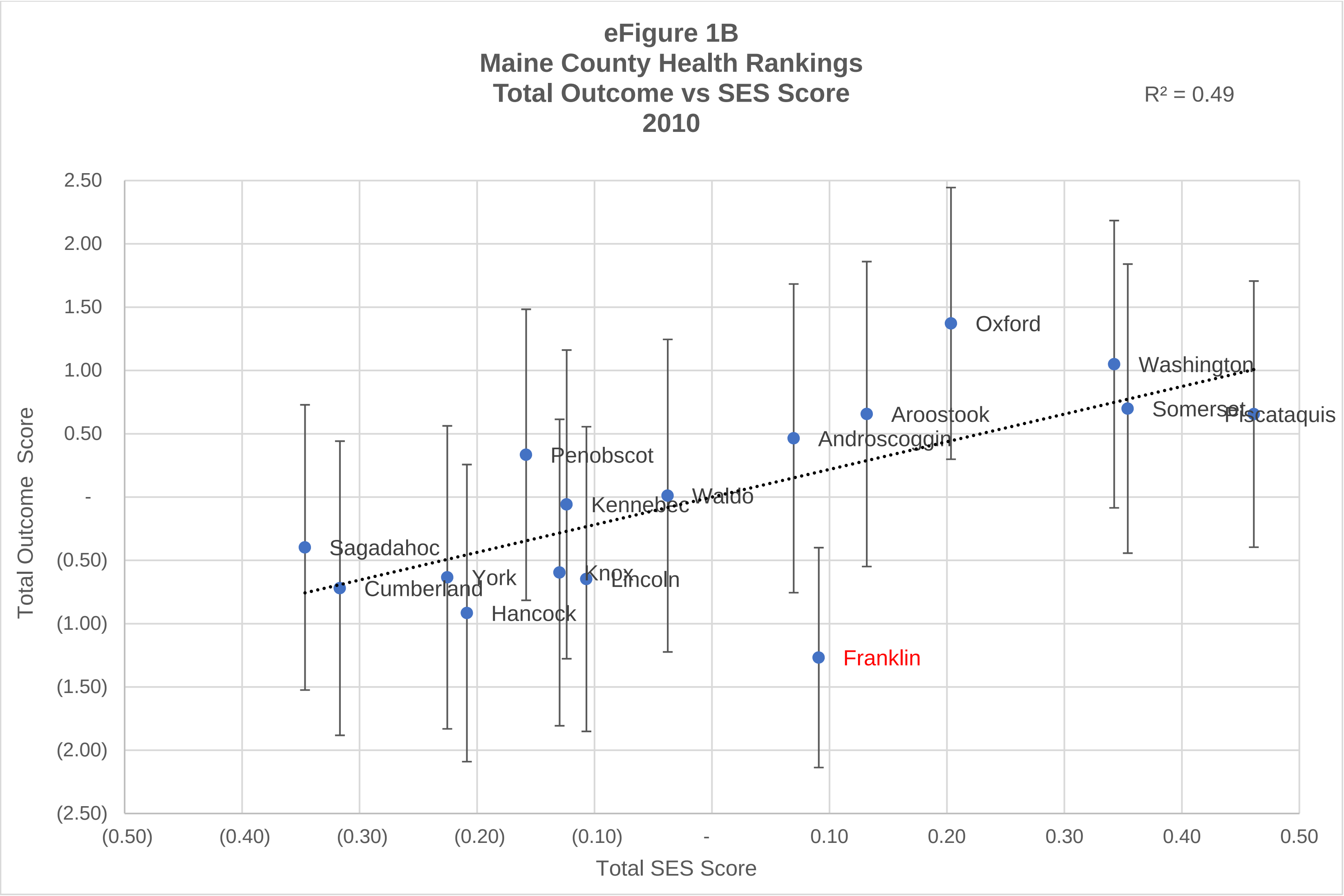

Legend for eFigure 1B,C:Maine county data for summary scores Total Outcome and Total Socioeconomic Score for each year were obtained from the County Health Rankings project (University of Wisconsin Population Health Institute and Robert Wood Johnson Foundation 2018). Years 2010 and 2018 were chosen because much of the data in CHR is retrospective from periods earlier than 2018 and reflects the earlier periods. Linear regression was performed and regression R^2 were calculated. Scatter plots of Total Outcome versus Socioeconomic Score of Maine counties for the years 2010 and 2018, and regression R^2 values for each year are shown.

© 2019 Onion DK et al. *JAMA Network Open.*

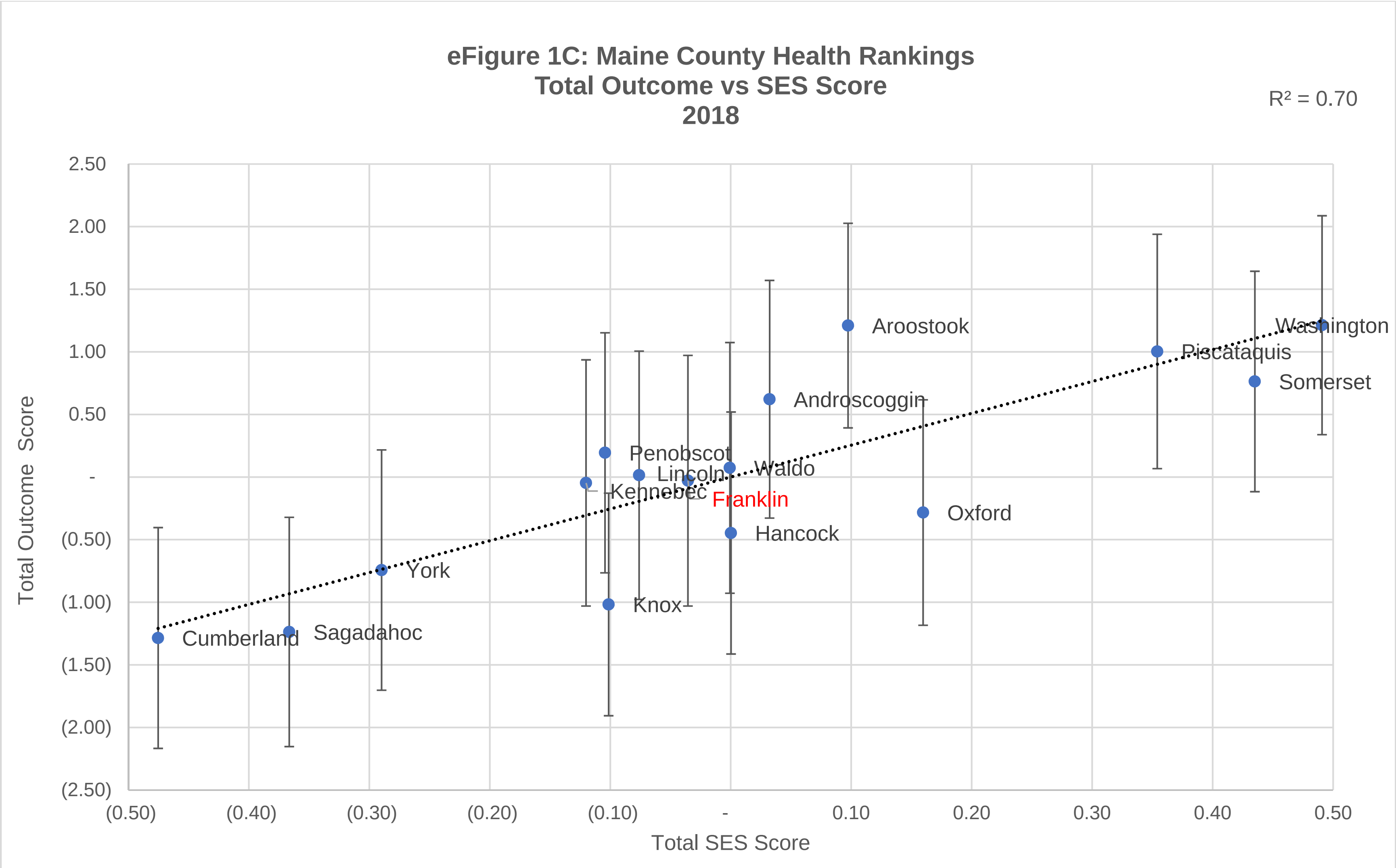

Legend for eFigure 1B,C:Maine county data for summary scores Total Outcome and Total Socioeconomic Score for each year were obtained. Years 2010 and 2018 were chosen because much of the data in CHR is retrospective from periods earlier than 2018 and reflects the earlier periods. Linear regression was performed and regression R^2 were calculated. Scatter plots of Total Outcome versus Socioeconomic Score of Maine counties for the years 2010 and 2018, and regression R^2 values for each year are shown.

© 2019 Onion DK et al. *JAMA Network Open.*

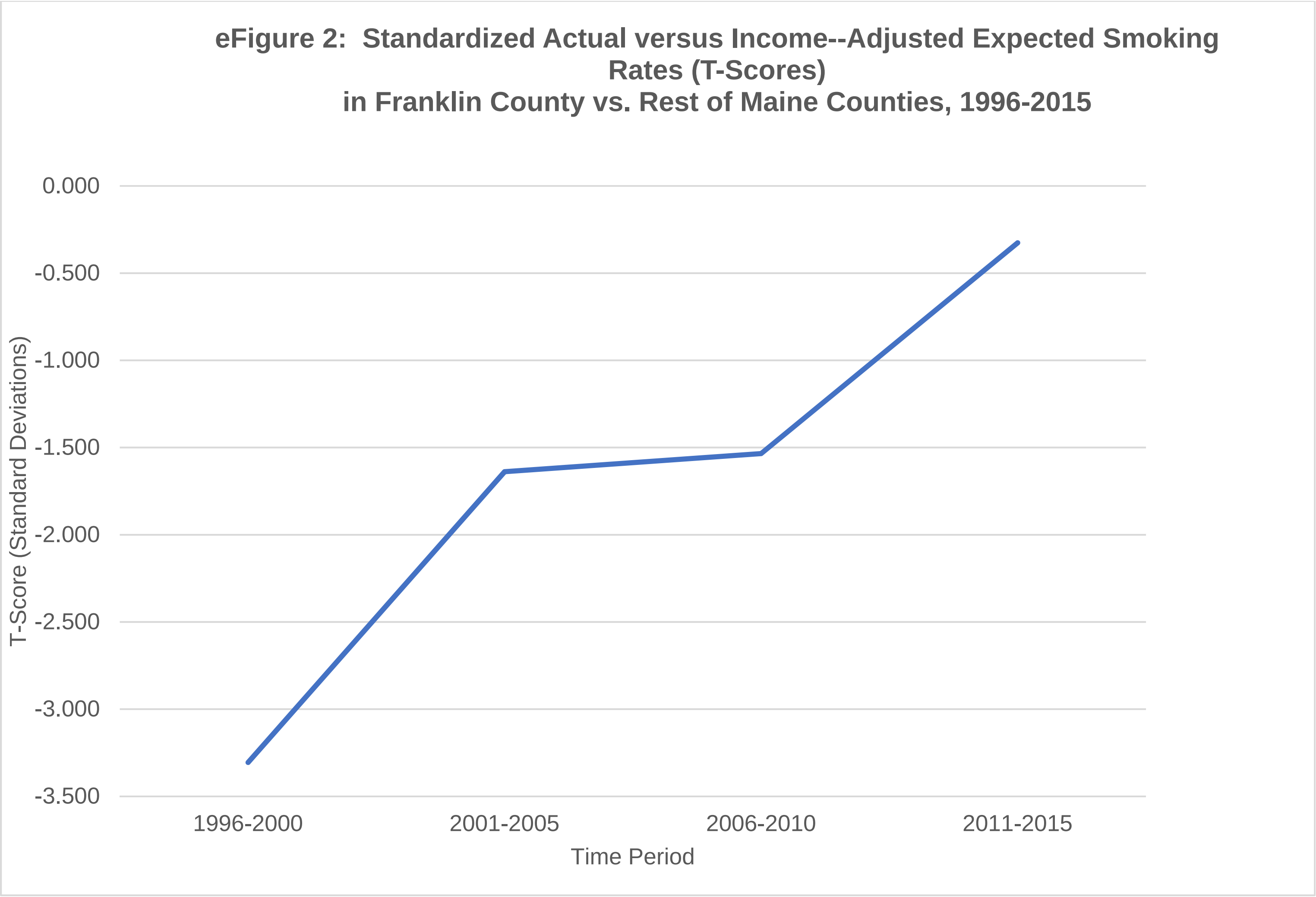

Legend for efigure 2: Mean present smoking rates for 5-year periods between 1996 and 2015 for Maine counties were obtained from the CDC Behavioral Risk Factor Surveillance System (United States Centers for Disease Control 1996-2015). Regression and analysis of variance between Maine county smoking rates were performed. County T scores how the number of standard deviations that the observed smoking rate differs from that predicted based on income. Positive T scores represent smoking rates greater than expected, and negative T scores represent rates less than expected. The Franklin County scores are shown.

© 2019 Onion DK et al. *JAMA Network Open.*

eFigure 3: Maine County Smoking Rates vs Income; Association R^2 Value

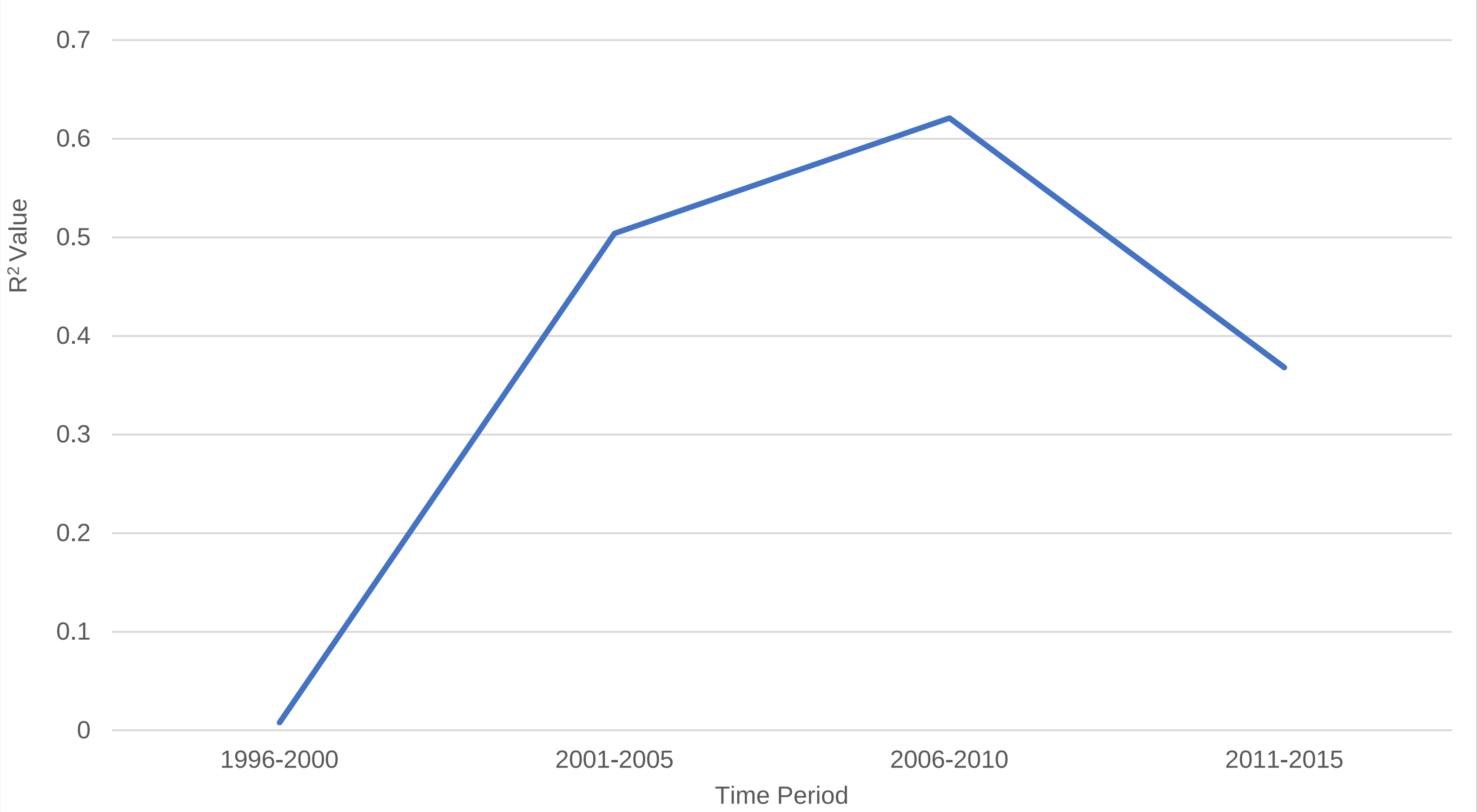

Legend for eFigure 3: Mean present smoking rates for 5-year periods between 1996 and 2015 for Maine counties were obtained from the CDC Behavioral Risk Factor Surveillance System (United States Centers for Disease Control 1996-2015). Regression and analysis of variance between Maine county smoking rates were performed. County T scores show the number of standard deviations that the observed smoking rate differs from that predicted based on income. Positive T scores represent smoking rates greater than expected, and negative T scores represent rates less than expected. The Franklin County scores are shown.

The R^2 values represent the proportion of the variance among Maine county smoking rates for each period that is associated with income. The apparent decrease in the relation between county income and smoking rate may be explained by the addition of cell phones as well as land line to the BRFSS survey, and the earlier sharp rise in the association may be partially explained by demographic changes in the household maintenance of land-line telephones. However same-period variations among counties seem to remain valid.

© 2019 Onion DK et al. *JAMA Network Open.*

Bibliography

United States Centers for Disease Control. 1996-2015. *Behavioral Risk Factor Surveillence System.* Vols. BRFSS Annual Data, 1996-2015. Atlanta, GA: United States Centers for Disease Control. Accessed December 11, 2018. https://www.cdc.gov/brfss/annual_data/annual_data.htm.

University of Wisconsin Population Health Institute and Robert Wood Johnson Foundation. 2018. *County Health Rankings & Roadmaps.* Madison, WI: University of Wisconsin Population Health Institute. Accessed December 10, 2018. http://www.countyhealthrankings.org/explore-health-rankings/rankings-data-documentation .

Whittington, John. 2013. *Counties of interest: achieving better- or worse-than-expected health outcomes: IHI 90-Day R&D Project Final Summary Report.* Cambridge, MA: Institute for Healthcare Improvement. http://www.ihi.org/knowledge/Pages/Publications/CountiesofInterestHealthOutcomesIHI90DayRDProject.aspx.

© 2019 Onion DK et al. *JAMA Network Open.*
